# GC-Content Normalization for RNA-Seq Data

**DOI:** 10.1186/1471-2105-12-480

**Published:** 2011-12-17

**Authors:** Davide Risso, Katja Schwartz, Gavin Sherlock, Sandrine Dudoit

**Affiliations:** 1 Department of Statistical Sciences, University of Padua, Italy; 2 Department of Genetics, Stanford University, USA; 3 Division of Biostatistics and Department of Statistics, University of California, Berkeley, USA

## Abstract

**Background:**

Transcriptome sequencing (RNA-Seq) has become the assay of choice for high-throughput studies of gene expression. However, as is the case with microarrays, major technology-related artifacts and biases affect the resulting expression measures. Normalization is therefore essential to ensure accurate inference of expression levels and subsequent analyses thereof.

**Results:**

We focus on biases related to GC-content and demonstrate the existence of strong sample-specific GC-content effects on RNA-Seq read counts, which can substantially bias differential expression analysis. We propose three simple within-lane gene-level GC-content normalization approaches and assess their performance on two different RNA-Seq datasets, involving different species and experimental designs. Our methods are compared to state-of-the-art normalization procedures in terms of bias and mean squared error for expression fold-change estimation and in terms of Type I error and *p*-value distributions for tests of differential expression. The exploratory data analysis and normalization methods proposed in this article are implemented in the open-source Bioconductor R package EDASeq.

**Conclusions:**

Our within-lane normalization procedures, followed by between-lane normalization, reduce GC-content bias and lead to more accurate estimates of expression fold-changes and tests of differential expression. Such results are crucial for the biological interpretation of RNA-Seq experiments, where downstream analyses can be sensitive to the supplied lists of genes.

## Background

In the last few years, high-throughput sequencing assays have been replacing microarrays as the assays of choice for measuring genome-wide transcription levels, in so-called *RNA-Seq *[1,2], as well as DNA copy number (*DNA-Seq*), protein-nucleic acid interactions (*ChIP-Seq*), and DNA methylation (*methyl-Seq *and *RRBS*). Several studies assessing technical aspects of RNA-Seq have shown good reproducibility and significant improvements over microarrays in terms of dynamic range and accuracy of expression fold-change estimation [3-5]. Nonetheless, as with microarrays, major technology-related artifacts and biases affect the expression measures [3,6-20] and normalization remains an important issue, despite initial optimistic claims such as: "One particularly powerful advantage of RNA-Seq is that it can capture transcriptome dynamics across different tissues or conditions without sophisticated normalization of data sets" [2].

Here, we focus on biases related to GC-content in the context of RNA-Seq data generated using the Illumina Genome Analyzer platform. Briefly, mRNA is converted to cDNA fragments which are then sequenced to produce millions of short *reads *(typically 25-100 bases). These reads are then mapped back to a reference genome and the number of reads mapping to a particular gene reflects the abundance of the transcript in the sample of interest. However, raw counts are neither directly comparable between genes within a lane, nor between replicate lanes (i.e., lanes assaying the same library) for a given gene, and normalization of the counts is needed to allow accurate inference of differences in transcript levels. Indeed, by virtue of the assay, one expects the read count for a given gene to be roughly proportional to both the gene's length and its transcript abundance. The read count will also vary between replicate lanes as a result of differences in *sequencing depth*, i.e., total number of reads produced in a given lane.

Furthermore, as detailed in the literature review below, previous studies have reported selection biases related to the sequencing efficiency of genomic regions, whereby read counts depend not only on length but also on sequence features such as GC-content and mappability (i.e., uniqueness of a particular sequence compared to the rest of the genome) [3,6-20]. For instance, GC-rich and GC-poor fragments tend to be under-represented in RNA-Seq, so that, within a lane, read counts are not directly comparable between genes. Additionally, GC-content effects tend to be lane-specific, so that the read counts for a given gene are not directly comparable between lanes. Biases related to length and GC-content confound differential expression (DE) results as well as downstream analyses, such as those involving Gene Ontology (GO). As GC-content varies throughout the genome and is often associated with functionality, it may be difficult to infer true expression levels from biased read count measures. Proper normalization of read counts is therefore crucial to allow accurate inference of differences in expression levels.

Herein, we distinguish between two main types of effects on read counts: (1) within-lane gene-specific (and possibly lane-specific) effects, e.g., related to gene length or GC-content, and (2) effects related to between-lane distributional differences, e.g., sequencing depth. Accordingly, *within-lane *and *between-lane normalization *adjust for the first and second types of effects, respectively.

### Within-lane normalization

The most obvious and well-known selection bias in RNA-Seq is due to *gene length*. Bullard *et al*. [3] and Oshlack & Wakefield [14] show that scaling counts by gene length is not sufficient for removing this bias and that the power of common tests of differential expression is positively correlated with both gene length and expression level. Indeed, the longer the gene, the higher the read count for a given expression level; thus, any method for which precision is related to read count will tend to report more significant DE statistics for longer genes, even when considering per-base read counts. Hansen *et al*. [12] incorporate length effects on the mean of a Poisson model for read counts using natural cubic splines and adjust for this effect using robust quantile regression. Young *et al*. [19] propose a method that accounts for gene length bias in Gene Ontology analysis after performing DE tests.

Another documented source of bias for the Illumina sequencing technology is *GC-content*, i.e., the proportion of G and C nucleotides in a region of interest. Several authors have reported strong GC-content biases in DNA-Seq [7,10] and ChIP-Seq [17]. Yoon *et al*. [18] propose a GC-content normalization method for DNA copy number studies, which involves binning reads in 100-bp windows and scaling bin-level read counts by the ratio between the overall median and the median for bins with the same GC-content. More recently, Boeva *et al*. [8] propose a polynomial regression approach, based on binning reads in non-overlapping windows and regressing bin-level counts on GC-content (with default polynomial degree of three). Still in the context of DNA-Seq, Benjamini & Speed [6] report that read counts are most affected by the GC-content of the actual DNA fragments from the sequence library (vs. that of the sequenced reads themselves) and that the effect of GC-content is sample-specific and unimodal, i.e., both GC-rich and GC-poor fragments are under-represented. They develop a method for estimating and correcting for GC-content bias that works at the base-pair level and accommodates library, strand, and fragment length information, as well as varying bin sizes throughout the genome.

Sequence composition biases have also been observed in RNA-Seq. Hansen *et al*. [11] report large and reproducible base-specific read biases associated with random hexamer priming in Illumina's standard library preparation protocol. The bias takes the form of patterns in the nucleotide frequencies of the first dozen or so bases of a read. They provide a re-weighting scheme, where each read is assigned a weight based on its nucleotide composition, to mitigate the impact of the bias and improve the uniformity of reads along expressed transcripts.

Roberts *et al*. [16] also consider the problem of non-uniform cDNA fragment distribution in RNA-Seq and use a likelihood-based approach for correcting for this fragment bias.

When analyzing RNA-Seq data from a yeast diploid hybrid for allele-specific expression (ASE), Bullard *et al*. [9] note that read counts from an orthologous pair of genes might overestimate the expression level of the more GC-rich ortholog. To correct for this confounding effect, they develop a resampling-based method where the significance of differences in read counts is assessed by reference to a null distribution that accounts for between-species differences in nucleotide composition.

While there has been general agreement about the need to adjust for GC-content effects when comparing read counts *between genomic regions for a given sample *(as in DNA-Seq and ChIP-Seq) or between orthologs (as in ASE with RNA-Seq in an F1 hybrid organism [9]), the need to do so was not immediately recognized for standard RNA-Seq DE studies, where one compares read counts *between samples for a given gene*. The common belief was that, for a given gene, the GC-content effect was the same across samples and hence would cancel out when considering DE statistics such as count ratios. Pickrell *et al*. [15] seem to be the first to note the sample-specificity of the GC-content effect in the context of RNA-Seq and the resulting confounding of expression fold-change estimates. To address this problem, they developed a lane-specific correction procedure which involves binning exons according to GC-content, defining for each GC-bin and each lane a relative read enrichment factor as the proportion of reads in that bin originating from that lane divided by the overall proportion of reads in that lane, and scaling exon-level counts by the spline-smoothed enrichment factors. As noted by Hansen *et al*. [12], this approach suffers from two main drawbacks. Firstly, as the enrichment factors are computed for each lane relative to all others, the procedure equalizes the GC-content effect across lanes instead of removing it. Secondly, by adding counts across exons and lanes, the method does not account for the fact that regions with higher counts also tend to have higher variances.

Zheng *et al*. [20] note that base-level read counts from RNA-Seq may not be randomly distributed along the transcriptome and can be affected by local nucleotide composition. They propose an approach based on generalized additive models to simultaneously correct for different sources of bias, such as gene length, GC-content, and dinucleotide frequencies.

In their recent manuscript, Hansen *et al*. [12] show that GC-content has a strong impact on expression fold-change estimation and that failure to adjust for this effect can mislead differential expression analysis. They develop a conditional quantile normalization (CQN) procedure, which combines both within and between-lane normalization and is based on a Poisson model for read counts. Lane-specific systematic biases, such as GC-content and length effects, are incorporated as smooth functions using natural cubic splines and estimated using robust quantile regression. In order to account for distributional differences between lanes, a full-quantile normalization procedure is adopted, in the spirit of that considered in Bullard *et al*. [3]. The main advantage of this approach is that it is lane-specific, i.e., it works independently in each lane, aiming at removing the bias rather than equalizing it across lanes. Modeling simultaneously GC-content and length (and in principle other sources of bias) leads to a flexible normalization method. On the other hand, for some datasets such as the Yeast dataset analysed in the present article, a regression approach may be too weak to completely remove the GC-content effect and other more aggressive normalization strategies may be needed.

### Between-lane normalization

The simplest between-lane normalization procedure adjusts for lane sequencing depth by dividing gene-level read counts by the total number of reads per lane (as in multiplicative Poisson model of Marioni *et al*. [4] and Reads Per Kilobase of exon model per Million mapped reads (RPKM) of Mortazavi *et al*. [5]). However, this still widely-used approach has proven ineffective and more beneficial procedures have been proposed [3,12,21,22].

In particular, Bullard *et al*. [3] consider three main types of between-lane normalization procedures: (1) *global-scaling *procedures, where counts are scaled by a single factor per lane (e.g., total count as in RPKM, count for housekeeping gene, or single quantile of count distribution); (2) *full-quantile *(FQ) normalization procedures, where all quantiles of the count distributions are matched between lanes; and (3) procedures based on *generalized linear models *(GLM). They demonstrate the large impact of normalization on differential expression results; in some contexts, sensitivity varies more between normalization procedures than between DE methods. Standard total-count normalization (cf. RPKM) tends to be heavily affected by a relatively small proportion of highly-expressed genes and can lead to biased DE results, while the upper-quartile (UQ) or full-quantile normalization procedures proposed in [3] tend to be more robust and improve sensitivity without loss of specificity.

In this article, we propose three different strategies to normalize RNA-Seq data for GC-content following a *within-lane *(i.e., sample-specific) gene-level approach. We examine their performance on two different types of data: a new RNA-Seq dataset for yeast grown in three different media and well-known benchmarking RNA-Seq datasets for two types of human reference samples from the MicroArray Quality Control (MAQC) Project [23]. For the latter datasets, the gene expression measures from qRT-PCR and Affymetrix chips serve as useful standards for performance assessment of RNA-Seq. We compare our approaches to the state-of-the-art CQN procedure of Hansen *et al*. [12] (which was shown to outperform competing methods such as that of Pickrell *et al*. [15]), in terms of bias and mean squared error for expression fold-change estimation and in terms of Type I error and *p*-value distributions for tests of differential expression. We demonstrate how properly correcting for GC-content bias, as well as for between-lane differences in count distributions, leads to more accurate estimation of gene expression levels and fold-changes, making statistical inference of differential expression less prone to false discoveries. The exploratory data analysis and normalization methods proposed in this article are implemented in the open-source Bioconductor R package EDASeq.

## Methods

### Data

We benchmark our proposed normalization methods on two different types of data: a new RNA-Seq dataset for yeast grown in three different media and the MAQC RNA-Seq datasets. The Yeast dataset addresses a "real" biological question, while the MAQC datasets are rather "artificial", but have the advantage of including qRT-PCR and Affymetrix chip measures for comparison with RNA-Seq. The different experimental designs allow the study of different types of technical and biological effects.

By *technical replicate lanes*, we refer to lanes assaying libraries that differ only by virtue of the sequencing assay (i.e., library preparation, flow-cell, lane), not in terms of the biology (i.e., growth condition or culture for the Yeast dataset, UHR vs. Brain for the MAQC-2 dataset). By *biological replicate lanes*, we refer to lanes assaying libraries that are distinct independently of/prior to the sequencing assay (i.e., libraries Y1, Y2, Y4, and Y7, for different cultures of the same yeast strain under the same growth condition for the Yeast dataset). There are therefore different levels/types of technical replication, depending on which aspect of the assay is varied (i.e., library preparation, flow-cell, lane). Likewise, there are different levels/types of biological replication. Furthermore, it is possible for biological effects to be *confounded *with technical effects, as is the case with culture and library preparation effects for the Yeast dataset.

The MAQC datasets are useful mainly for examining technical effects, i.e., for understanding the biases and variability introduced at various stages of the assay, as was done in Bullard *et al*. [3]. The Yeast dataset allows the study of both technical and biological effects of interest.

#### Yeast dataset

Illumina's Genome Analyzer II high-throughput sequencing system was used to sequence RNA from *Saccharomyces cerevisiae *grown in three different media: standard YP Glucose (YPD, a rich medium), Delft Glucose (Del, a minimal medium), and YP Glycerol (Gly, which contains a non-fermentable carbon source in which cells respire rather than ferment). Specifically, yeast (diploid S288c) were grown at 25°C to approximately 1-2*e*7 cells/ml, as determined by a Beckman Coulter Z2 Particle Count and Size Analyzer. Cells were harvested by filtration, frozen in liquid nitrogen, and kept at -80°C until RNA extraction and purification. RNA was extracted from the cells using a slightly modified version of the traditional hot phenol protocol [24], followed by ethanol precipitation and washing. Briefly, 5 ml of lysis buffer (10 mM EDTA pH 8.0, 0.5% SDS, 10 mM Tris-HCl pH 7.5) and 5 ml of acid phenol were added to frozen cells and incubated at 60°C for 1 hour, with occasional vortexing, then placed on ice. The aqueous phase was extracted after centrifuging and additional phenol extraction steps were performed as needed, followed by a chloroform extraction. Total RNA was precipitated from the final aqueous solution, with 10% volume 3 M sodium acetate pH 5.2 and ethanol, and resuspended in nuclease-free water. Residual DNA was removed from the RNA preparations using the Turbo DNA-free kit (Applied Biosystems/Ambion, AM1907). PolyA RNA was prepared using the Poly(A)Purist MAG kit (Applied Biosystems/Ambion, AM1922). Strand-specific RNA-Seq libraries were prepared starting with 1-2 *μ*g of polyA RNA using two different protocols [25,26]. "Protocol 1" follows Maniar & Fire [25], as described, and "Protocol 2" follows Parkhomchuk *et al*. [26], as in [27] with the following modifications: fragmentation was carried out before cDNA synthesis as above and gel purification after PCR amplification was omitted.

The experimental design for the Yeast dataset is summarized in Table 1. Four distinct colonies were used to inoculate independent YPD cultures (Y1, Y2, Y4, and Y7), each yielding a single RNA library, which was then sequenced using two lanes of possibly different flow-cells. The libraries for Y1, Y2, and Y7 were prepared using Protocol 1 and the library for Y4 was prepared using Protocol 2. For the Delft medium, there are three cultures, each sequenced using Protocol 1 on one lane within the same flow-cell. For the Glycerol medium, there are also three cultures; culture G1 was sequenced in a single lane using Protocol 2, while cultures G2 and G3 were each sequenced using Protocol 1 and one lane of the same flow-cell (distinct from that of G1).

**Table 1:** Yeast dataset: Experimental design

	**Culture/Library prep**.	Library prep. protocol	Growth condition	Flow-cell
1	Y1	Protocol 1	YPD	428R1
2	Y1	Protocol 1	YPD	4328B
3	Y2	Protocol 1	YPD	428R1
4	Y2	Protocol 1	YPD	4328B
5	Y7	Protocol 1	YPD	428R1
6	Y7	Protocol 1	YPD	4328B
7	Y4	Protocol 2	YPD	61MKN
8	Y4	Protocol 2	YPD	61MKN
9	D1	Protocol 1	Del	428R1
10	D2	Protocol 1	Del	428R1
11	D7	Protocol 1	Del	428R1
12	G1	Protocol 2	Gly	6247L
13	G2	Protocol 1	Gly	62OAY
14	G3	Protocol 1	Gly	62OAY

With three growth conditions and ten cultures from independent colonies sequenced using two different library preparation protocols and either one or two lanes in a total of five flow-cells, the design allows us to examine both technical effects (e.g., library preparation, flow-cell, lane) and biological effects (e.g., growth condition, culture). Cultures grown under the same condition are viewed as biological replicates (i.e., Y1, Y2, Y4, and Y7). There are various levels of technical replication: library preparation protocol, library preparation (with same protocol), flow-cell, lane. Note, however, that here library preparation (technical) effects are confounded with culture (biological) effects.

Illumina's standard Genome Analyzer pre-processing pipeline was used to yield 36 bp-long single-end reads. Reads were mapped to the reference genome [28] using Bowtie [29], considering only unique mapping and allowing up to two mismatches (Figures S1 and S2, Additional File 1). The read count for a given gene is defined as the number of reads with 5'-end falling within the corresponding region. Genes with an average read count below 10 for each of the three growth conditions were filtered out, i.e., gene *j *was filtered out if $\max_{k\in \left\{\text{YPD,Del,Gly}\right\}}{ȳ}_{j,k}<10$, where ${ȳ}_{j,k}$ denotes the average read count for gene *j *in condition *k*. This procedure retained 5,690 (out of 6,575) genes.

The Yeast data are available in the NCBI’s Sequence Read Archive (SRA) [http://www.ncbi.nlm.nih.gov/sra], under the accession number SRA048710.

#### MAQC datasets

Illumina's Genome Analyzer II high-throughput sequencing system was used to sequence RNA for two types of biological samples from the MicroArray Quality Control (MAQC) Project [23]: Ambion's human brain reference RNA ("Brain"), pooled from multiple donors and several brain regions, and Stratagene's universal human reference RNA ("UHR"), a mixture of total RNA extracted from 10 different human cell lines. The data are summarized below; additional detail about experimental design, pre-processing, and the associated qRT-PCR and microarray datasets can be found in Bullard *et al*. [3].

In dataset MAQC-2, Brain and UHR RNA were sequenced each using a single library preparation and seven lanes distributed across two flow-cells (i.e., technical replicates). There is no biological replication, but various types of technical replication (i.e., flow-cell, lane). Library preparation effects are confounded with the extreme differential expression one expects when comparing such different samples as Brain and UHR. Nonetheless, the availability of qRT-PCR measures for a subset of circa 1,000 genes makes this a valuable benchmarking dataset.

In dataset MAQC-3, four different library preparations of UHR RNA were each sequenced using three or four lanes from only one of two flow-cells. There is again no biological replication, but one can use this dataset for examining technical effects such as library preparation and lane effects. However, library preparations are nested within flow-cells, so that differences between flow-cells are confounded with library preparation effects.

For both the MAQC-2 and MAQC-3 datasets, reads were mapped to the genome (GRCh37 assembly) using Bowtie [29], with unique mapping and up to two mismatches. Gene-level counts were obtained using the *union-intersection *(UI) gene model of [3]. Low-count genes were filtered out using a procedure analogous to that used for the Yeast dataset. Specifically, for MAQC-2, genes with an average read count below 10 for both the Brain and UHR samples were filtered out, yielding 12,340 (out of 39,359) genes. For MAQC-3, genes with an average read count below 10 for each of the four libraries were filtered out, yielding 11,847 (out of 39,359) genes.

In the original MAQC paper [23], 997 genes were assayed by qRT-PCR, with four measures (i.e., technical replicates) for each of the Brain and UHR samples. This technology is regarded as yielding accurate estimates of expression levels and is used here as a gold standard for comparing normalization methods. Following [3], we consider only the genes which match a unique UI gene, are called present in at least three out of the four Brain and UHR runs, and have standard errors across the eight runs not exceeding 0.25. We found 638 genes in common with the RNA-Seq filtered genes and use this subset to compare expression measures between the technologies. The UHR/Brain expression log-fold-change of a gene is estimated by the log-ratio between the average of the four UHR measures and the average of the four Brain measures.

Moreover, as reported in [23], a number of microarray experiments were conducted on the Brain and UHR samples. As in [3], we consider the Affymetrix data from the first site, where each biological sample was assayed using five chips (i.e., technical replicates, GeneChip Human Genome U133 Plus 2.0 Array). We pre-processed the data using RMA [30], as implemented in the Bioconductor R package affy, and obtained *p*-values for UHR vs. Brain differential expression using the limma package [31], with the standard *lmFit *and *eBayes *pipeline. There are 11,081 genes detected by RNA-Seq and present on the Affymetrix chip. The MAQC data are available in the Sequence Read Archive, under the accession number SRA010153.

### Within-lane GC-content normalization

We propose three novel within-lane normalization approaches to account for the dependence of read counts on GC-content. The first method is based on the simple idea of regressing gene-level counts on GC-content and is implemented using the loess robust local *regression *procedure; the *global-scaling *and *full-quantile *normalization methods involve stratifying genes in equally-sized *bins *(i.e., bins containing the same number of genes) based on GC-content and then "matching" parameters of the count distributions across bins.

We choose to normalize the logarithms of the gene-level counts for at least two reasons. Firstly, the logarithm is the canonical link for the Poisson (and negative binomial) distribution, hence it seems natural to work on the log-scale when considering regression for count data. Moreover, regression on the log-scale is more robust to the presence of outliers (i.e., extremely high counts) that can bias the fit.

In what follows, let *y_j _*and *x_j _*denote, respectively, the logarithm of the read count and the GC-content (i.e., proportion of G and C nucleotides in the gene sequence) for gene *j *= 1, ..., *J*.

#### Regression normalization

Gene-level read counts (log-scale) *y_j _*are regressed on GC-content *x_j _*using the loess robust local regression method [32] and normalized expression measures $y_{j}^{\prime }$ are obtained by shifting the residuals to recover the scale of the raw counts, i.e.,

(1) $$y_{j}^{\prime }=y_{j}-{ŷ}_{j}+T\left(y_{1},\;.\;.\;.\;,y_{J}\right),$$

where ${ŷ}_{j}$ denote the fitted values and *T *a summary statistic such as the median.

#### Global-scaling normalization

Genes are stratified into *K *equally-sized bins based on GC-content. The normalized expression measures are defined as

(2) $$y_{j}^{\prime }=y_{j}-T\left(y_{j^{\prime }}:j^{\prime }\in k\left(j\right)\right)+T\left(y_{1},\;.\;.\;.,y_{J}\right),$$

where *k*(*j*) denotes the GC-content stratum to which gene *j *belongs and *T *denotes a summary statistic, e.g., median, upper-quartile, or count for control genes. For instance, on the original (unlogged) scale, the normalized count for a particular gene could be its raw count divided by the ratio of the median count in its GC-bin to the overall median count of all genes.

#### Full-quantile normalization

In full-quantile (FQ) normalization, genes are stratified according to GC-content as for global-scaling normalization. The quantiles of the read count distributions are then matched between GC-bins, by sorting counts within bins and then taking the median of quantiles across bins. This approach is analogous to the microarray between-chip normalization of Irizarry *et al*. [30] and the RNA-Seq between-lane normalization of Bullard *et al*. [3].

### Between-lane normalization

GC-content normalization is designed to reduce the dependence of gene-level read counts on sequence composition *within *a lane. However, other technical effects, such as between-lane differences in sequencing depth, can strongly bias differential expression results. We therefore apply a *between-lane *normalization procedure, as in Bullard *et al*. [3], after *within-lane *normalization and before differential expression analysis.

Between-lane normalization methods inherently make count distributions more similar between lanes, at the risk of dampening down true differential expression. Full-quantile normalization is the most aggressive of the methods we have proposed and both FQ and total-count (cf. RPKM) normalization force equal library sizes (i.e., total counts) across lanes. For the Yeast dataset, all four between-lane normalization methods (global-scaling normalization with total-count, upper-quartile, and median, and full-quantile normalization) appear to yield similar results (data not shown). Since the CQN approach of Hansen *et al*. [12] involves FQ between-lane normalization, we settle on FQ normalization for comparison purposes (Figure S2). Such a between-sample normalization approach was used for microarrays in Irizarry *et al*. [30]. A thorough study of between-lane normalization procedures is beyond the scope of this paper and was carried out in Bullard *et al*. [3].

### Implementation of within and between-lane normalization procedures

The above within-lane and between-lane normalization procedures are implemented in the functions withinLaneNormalization and betweenLaneNormalization, respectively, of the EDASeq package. For GC-content normalization, the withinLaneNormalization function takes as input a genes-by-lanes table of counts and a vector of gene GC-content values and returns a genes-by-lanes table of normalized counts, on the original unlogged scale and rounded to the nearest integer. There is also the option to output a table of normalization offsets, equal to the difference between the normalized and unnormalized counts. The normalized counts (with offset set to zero) or the unnormalized counts and corresponding offsets can then be supplied to standard R packages for differential expression analysis, such as DESeq [21] or edgeR [33]. Details are provided in the EDASeq package vignette and help pages.

### Differential expression analysis

*Differential expression *(DE) analysis is performed using *likelihood ratio tests *(LRT) based on a *negative binomial model *for gene-level read counts [21,33]. The negative binomial distribution can be viewed as an extension of the Poisson distribution, which accommodates *over-dispersion *by modeling the variance as a quadratic function of the mean *μ*, *V*(*μ*) = *μ *+ *ϕμ*^2^, with dispersion parameter *ϕ*. For *ϕ *= 0, one recovers the Poisson distribution.

We use the Bioconductor R package edgeR [33] to fit a negative binomial model to gene-level read counts and perform likelihood ratio tests of DE. A common dispersion parameter is estimated for all genes.

While Bullard *et al*. [3] found that the Poisson distribution was appropriate for the MAQC datasets (indeed, the edgeR estimates of the dispersion parameters are near zero), we have noticed substantial over-dispersion for the Yeast dataset, even after between-lane normalization ($\hat{\phi }=0.078$, Figure S3).

### Evaluation criteria

Our aim is to evaluate GC-content normalization approaches in terms of their impact on differential expression results. To achieve this, we consider bias and mean squared error in expression fold-change estimation. We also compare normalization methods in terms of their Type I error rates and *p*-value distributions for likelihood ratio tests of DE based on a negative binomial model for gene-level read counts [33].

The global-scaling and full-quantile within-lane normalization approaches were implemented using *K *= 10 GC-content bins for the MAQC datasets and *K *= 50 bins for the Yeast dataset, to reflect the strength of the GC-content effect for each dataset.

#### Bias and mean squared error for expression fold-change estimation

Expression log-fold-changes are estimated by log-ratios of average normalized read counts between two sets of lanes corresponding to the two conditions of interest.

In order to compute *bias *and *mean squared error *(MSE), one needs to know the true value of the expression fold-change. For the MAQC-2 dataset, one can use the estimate of the UHR/Brain fold-change from qRT-PCR as the true value, since qRT-PCR is often considered as a gold standard for producing accurate estimates of expression levels. The RNA-Seq estimated fold-change is the ratio of the average of the normalized counts for the seven UHR lanes to the average of the normalized counts for the seven Brain lanes. For a given gene, bias is then estimated as the difference between the estimated log-fold-changes from the two technologies.

For the Yeast dataset, we consider only the eight YPD lanes (Table 1), for which we do not expect any differential expression, and assume that the true log-fold-change when comparing any combination of such lanes is zero. Specifically, we consider all ${\text{(}}_{4}^{8}\text{)}/2=\text{35}$ possible combinations of the eight YPD lanes into two groups of four lanes each. For each such "null pseudo-dataset", we compute the log-ratio of average normalized read counts between the two groups of four lanes. For a given gene, bias is estimated as the average of these 35 log-ratios and MSE as the average of the square of these 35 log-ratios.

#### Testing DE based on negative binomial model

To evaluate the impact of normalization on differential expression results, we use the edgeR package [33] to perform gene-level likelihood ratio tests of DE, based on a negative binomial model for read counts, with common dispersion parameter.

For the Yeast dataset, we assess *Type I error *by considering again all 35 YPD null pseudo-datasets and by testing for DE between each of the corresponding two groups. Such a setting is intended to mimic the null hypothesis of no DE and any gene called DE yields a false positive. For a given pseudo-dataset and *nominal *Type I error rate *α*, the *actual *Type I error rate is defined as the proportion of genes with unadjusted *p*-values not exceeding *α*.

It is not possible to assess power with the Yeast dataset, as one cannot identify with certainty the set of all genes expected to be DE between growth conditions. Nonetheless, we perform gene-level tests of DE between the three growth conditions using edgeR and compare *p-value distributions *and numbers of genes declared DE between different normalization procedures.

For the MAQC samples, we compare UHR vs. Brain differential expression results based on Illumina RNA-Seq and Affymetrix chip data. For RNA-Seq, we perform tests of DE between the seven Brain and seven UHR lanes using edgeR. Tests of DE between the five Brain and five UHR chips are performed using limma. We then examine *p*-value distributions and numbers of genes declared DE for the two technologies.

## Results

### GC-content effect

**Figure 1:**
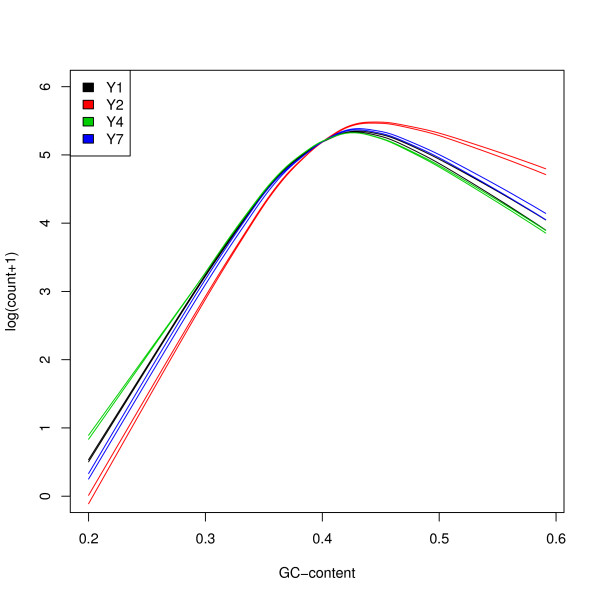
**Yeast dataset: Read count vs. GC-content**. Lowess fits of gene-level log(count + 1) vs. GC-content for the eight YPD lanes from the Yeast dataset, after FQ between-lane normalization. Curves are colored according to culture/library preparation. The GC-content effect is the same for lanes assaying the same culture/library preparation, but can be different for lanes assaying different cultures/library preparations. Figure S4 displays the scatterplot and lowess fit for the first YPD lane (culture/library preparation Y1, flow-cell 428R1).

As noted in Pickrell *et al*. [15] and Hansen *et al*. [12], the GC-content bias on read counts is *sample-specific*, meaning that the dependence of gene counts on GC-content may vary between lanes. For the Yeast dataset, Figures 1 and S4 show that the relationship between read count and GC-content (after between-lane normalization) is the same for lanes assaying the same culture/library preparation, but can be different for lanes assaying different cultures/library preparations. The GC-content effect is also *unimodal*, in the sense that read counts first increase, then decrease with GC-content. Likewise, for the MAQC datasets, Figure S5 illustrates that the GC-content effect varies between different library preparations, but not within library preparations, although the effect is weaker than for the Yeast dataset. These observations suggest that the GC-content bias is likely to be introduced at the library preparation step (although one should recall that for the Yeast dataset, culture and library preparation effects are confounded; see Table 1). Our findings are in agreement with Benjamini & Speed [6], who point to PCR as the most important cause for GC-content bias.

**Figure 2:**
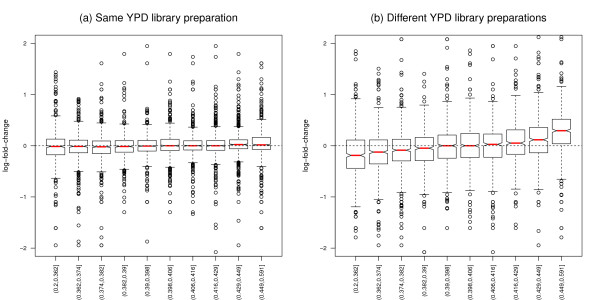
**Yeast dataset: Log-fold-change vs. GC-content**. Stratified boxplots of count log-ratio vs. GC-content, after FQ between-lane normalization. Panel (a): Same culture/library preparation, YPD Y1 lanes from flow-cells 428R1 vs. 4328B. Panel (b): Different cultures/library preparations, YPD Y1 lane vs. Y2 lane from flow-cell 428R1. The GC-content effect is the same for the two lanes assaying the same culture/library preparation, so that fold-change estimates do not vary with GC-content. By contrast, the GC-content effect differs between cultures/library preparations and confounds fold-change estimation.

The strong impact of GC-content on expression fold-change estimation is illustrated in Figures 2, S6, and S7, which contrast count log-ratios for lanes assaying the same library preparation to count log-ratios for lanes assaying different library preparations. Specifically, for the Yeast dataset, log-ratios for YPD lanes that are not expected to exhibit any DE do not depend on GC-content for the same culture/library preparation (Figure 2, Panel (a)), but increase monotonically with GC-content for two different cultures/library preparations (Panel (b)). For the MAQC-2 dataset, log-ratios for two lanes from the same UHR library preparation do not depend on GC-content (Figure S6, Panel (a)), while log-ratios for Brain and UHR lanes vary with GC-content (Panel (b)). For the MAQC-3 dataset, where one expects no differential expression, log-ratios for two lanes from the same UHR library preparation do not depend on GC-content (Figure S7, Panel (a)), while log-ratios for two lanes from different UHR library preparations do depend on GC-content (Panel (b)).

**Figure 3:**
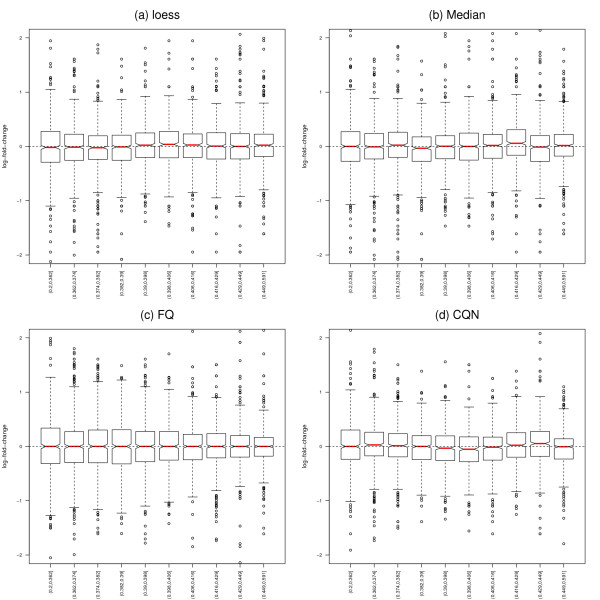
**Yeast dataset: GC-normalized log-fold-change vs. GC-content**. Stratified boxplots of count log-ratio vs. GC-content, for the two YPD cultures/library preparations of Figure 2, Panel (b), for four within-lane GC-content normalization procedures. Panel (a): Regression normalization using loess. Panel (b): Global-scaling normalization using the median. Panel (c): Full-quantile (FQ) normalization. Panel (d): Conditional quantile normalization (CQN). The first three within-lane procedures were followed by FQ between-lane normalization; CQN includes its own between-lane normalization. All methods seem to effectively reduce the dependence of fold-change on GC-content (compared to Figure 2, Panel (b)).

All four normalization procedures considered here reduce the dependence of both read counts and fold-change estimates on GC-content, with an edge for our proposed full-quantile normalization (Figures 3 and S8).

### Bias and mean squared error for expression fold-change estimation

**Figure 4:**
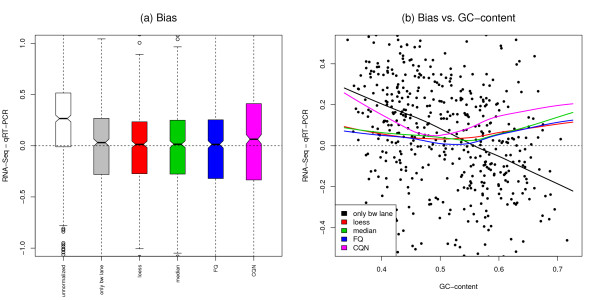
**MAQC-2 dataset: Bias in fold-change estimation**. Bias in UHR/Brain expression log-fold-change estimation for different RNA-Seq normalization procedures, where bias is defined as the difference between the estimates from RNA-Seq and qRT-PCR for 638 genes assayed by both technologies. Panel (a): Boxplots of bias in log-fold-change estimates. Our three proposed normalization procedures reduce bias, while CQN tends to overestimate the UHR/Brain fold-change. Panel (b): Dependence of bias on GC-content. The points correspond to bias after only FQ between-lane normalization, the curves are lowess fits of bias vs. GC-content for different normalization procedures. There is still substantial dependence of bias on GC-content after CQN.

For the MAQC-2 dataset, qRT-PCR may be viewed as a gold standard, so that bias for different RNA-Seq normalization procedures may be assessed based on differences in log-fold-change estimates from the two technologies. Figure 4, Panel (a), shows that most of the bias is due to differences in sequencing depths between lanes and that bias is greatly reduced by between-lane normalization. However, the black curve in Panel (b) indicates that with only between-lane normalization, there is a strong dependence of bias on GC-content. All four within-lane GC-content normalization procedures reduce bias and its dependence on GC-content, although CQN still tends to over-estimate fold-changes and is not as effective as the other three approaches in terms of removing the dependence of bias on GC-content.

Similar representations of bias and MSE are provided in Figures S9 and S10 for the Yeast YPD pseudo-datasets, for which one would expect the log-fold-changes to be around zero. All within-lane GC-content normalization methods perform similarly on these artificial data. Note, however, that fold-change estimates can vary greatly between the 35 datasets (Figure S11), likely due to culture/library preparation effects. There is a clear bias for unnormalized counts, with log-fold-change estimates as high as 2. The full-quantile GC-content normalization method seems to be the most coherent in estimating the log-fold-change around zero.

### Testing DE based on negative binomial model

#### Type I error rate

To assess the impact of normalization on DE tests, we first consider Type I error rates. For the Yeast data, the 35 YPD pseudo-datasets simulate an experiment in which all genes satisfy the null hypothesis of constant expression and hence any gene called DE is considered a false positive.

Figure S12 displays, for each of the 35 pseudo-datasets, the difference between the actual Type I error rate (i.e., the proportion of genes called DE) and the nominal Type I error rate for the negative binomial LRT implemented in the edgeR package [33]. The figure indicates that Type I error rates vary substantially between pseudo-datasets (likely due to culture/library preparation effects, as noted for Figure S11), although the median actual Type I error rate is close to the nominal value for all within-lane GC-content normalization methods.

**Figure 5:**
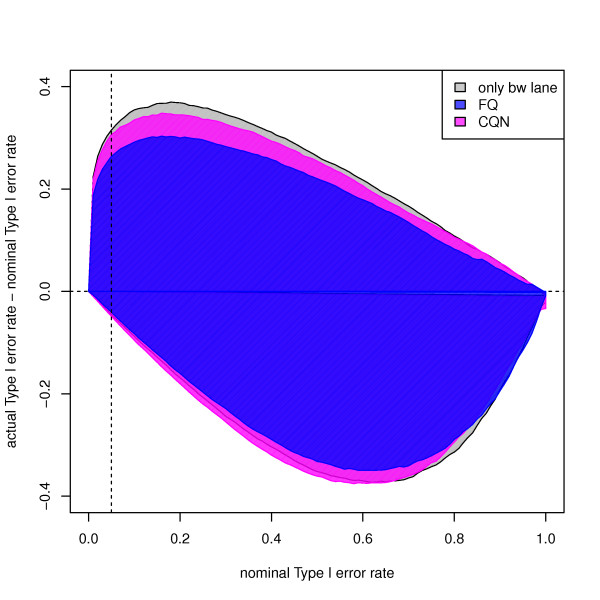
**Yeast YPD pseudo-datasets: Type I error**. Difference between actual and nominal Type I error rates vs. nominal Type I error rate, for different normalization procedures. The colored areas correspond to the most conservative and most anti-conservative curves obtained from the 35 YPD pseudo-datasets. The dashed line corresponds to a nominal unadjusted *p*-value of 0.05. The full-quantile GC-content normalization procedure yields the smallest area, meaning that the actual Type I error rate is closer to the nominal Type I error rate than with the other two procedures.

Figure 5 summarizes the Type I error rates for the 35 pseudo-datasets by the area between the curves corresponding to the most conservative and most anti-conservative behavior (i.e., "worst-case" scenario). Full-quantile GC-content normalization leads to the smallest area, indicating that the DE test is closer to its nominal level than with other procedures.

The 35 pseudo-datasets do not actually fully mimic the null hypothesis of no differential expression, due to culture/library preparation effects. Indeed, regardless of the normalization approach, and as expected from biology, the eight YPD lanes cluster according to culture. Only with median normalization does the clustering first reflect library preparation protocol (Figure S13). After verification, it turns out that the top curves in Figure 5 correspond to "imbalanced" pseudo-datasets, where lanes are split according to culture: Y1 (Protocol 1) and Y7 (Protocol 1) cultures in one group, Y2 (Protocol 1) and Y4 (Protocol 2) cultures in the other group. The analog of Figure 5 for the ${\text{(}}_{3}^{6}\text{)}/2=\text{1}0$ YPD pseudo-datasets for libraries prepared using Protocol 1 is provided in Figure S14. Interestingly, the difference between FQ within-lane normalization and only between-lane normalization becomes negligible, while CQN yields the most anti-conservative curve.

#### *p*-value distribution

**Figure 6:**
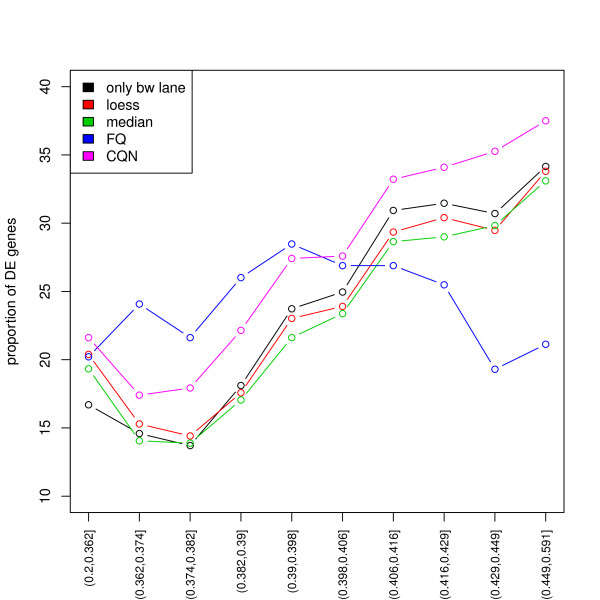
**Yeast dataset: Proportion of DE genes vs. GC-content**. Here, a gene is declared DE between the three growth conditions if its nominal unadjusted *p*-value from the negative binomial LRT is below the threshold of 10^-5 ^(corresponding to a nominal Bonferroni family wise error rate of 0.057 and Benjamini & Hochberg [37] false discovery rate of 4.22 × 10^-5^). There is a clear trend towards more detected differential expression at higher GC-content with all within-lane normalization procedures but the full-quantile.

To evaluate normalization methods in a biologically meaningful context, we consider the full Yeast dataset (i.e., all fourteen lanes) and perform gene-level LRT of growth condition effects on gene expression using edgeR. The stratified boxplots in Figure S15 reveal a clear dependence of *p*-values on GC-content for all but the full-quantile GC-content normalization method. Figure 6 indicates that the percentage of genes declared differentially expressed increases sharply with GC-content, again for all but full-quantile normalization (unadjusted *p*-value cut-off of 10^-5^). Similar results are observed with unadjusted *p*-value cut-offs of 0.01 and 0.001 (data not shown).

**Figure 7:**
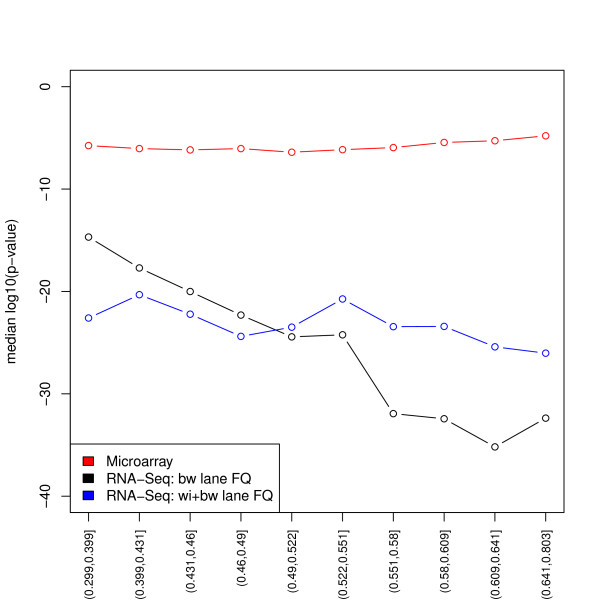
**MAQC-2 dataset: *p*-value vs. GC-content**. Median unadjusted *p*-value (log_10_) for each GC-content stratum, for microarray and RNA-Seq UHR vs. Brain DE analysis (11,081 genes detected by RNA-Seq and present on the Affymetrix chip). The figure shows that the GC-content bias is technology-related and that full-quantile within-lane normalization reduces the dependence of RNA-Seq *p*-values on GC-content.

For the MAQC-2 dataset, we examine *p*-value distributions when testing for DE between Brain and UHR using both RNA-Seq and Affymetrix chip data. Figure 7 shows that the GC-content effect on DE results is *technology-specific*. Indeed, for microarrays, *p*-values do not depend on GC-content. By contrast, with only between-lane normalization, RNA-Seq *p*-values tend to decrease with GC-content. Full-quantile within-lane GC-content normalization removes this dependence.

### Tuning parameters

The main tuning parameter in our proposed global-scaling and full-quantile GC-content normalization procedures is the *number of GC-content bins*. This parameter is analogous to the span in loess robust local regression, thus the same considerations of bias/variance trade-off should guide its selection. Intuitively, the larger the number of bins, the more adaptive and possibly noisy the normalization. The boxplots of bias in Figure S16 indicate that DE results are robust to the number of GC-content bins in FQ normalization. We used *K *= 10 bins for the MAQC datasets and *K *= 50 for the Yeast dataset; a selection which reflects the stronger GC-content effect observed for the latter dataset.

## Discussion

We have compared differential expression results based on our three proposed within-lane GC-content normalization methods and the CQN method of Hansen *et al*. [12], on the MAQC and Yeast datasets. Only full-quantile GC-content normalization appears to effectively remove the dependence of the proportion of DE genes on GC-content. This could mean either that, for some biological reason, GC-richer genes are more likely to be truly DE (in which case normalization erroneously removes this dependence) or that GC-content bias is so strong that an aggressive normalization method is needed. Since we are not aware of any plausible biological explanation for the dependence of DE results on GC-content, we believe that the MAQC and Yeast data require a full-quantile approach and that merely regressing counts on GC-content is not sufficient to completely remove the bias.

To rule out a biological reason for the dependence of DE on GC-content, we compared UHR vs. Brain DE results based on the MAQC-2 RNA-Seq data to those based on Affymetrix chip data [23]. Figure 7 clearly indicates that the dependence of *p*-values on GC-content is technology-specific, i.e., unlike RNA-Seq *p*-values, microarray *p*-values do not depend on GC-content. Full-quantile within-lane normalization reduces the dependence of *p*-values on GC-content. Interestingly, and encouragingly, the much smaller *p*-values for the RNA-Seq data suggest that this newer assay is more powerful than microarrays for DE analysis (although it is unclear how to relate numbers of lanes and numbers of chips in terms of sample size).

Another well-known selection bias in RNA-Seq is due to gene length [3,14]. For the Yeast dataset, we noticed only a minor length effect on read counts and DE results (Figure S17, Panel (a)). In fact, genes with high GC-content tend to be shorter, so there seems to be a compensation effect due to sequence composition (data not shown). Mappability does not appear to affect read counts for this dataset (Figure S17, Panel (b)). Gene length bias for the MAQC datasets is discussed in [3].

In addition to their good performance noted above, our proposed normalization methods offer a number of advantages. They are very simple to implement and extend and lead to DE results that are robust to tuning parameters such as the number of GC-content bins (Figure S16). They could be applied to other genomic regions (e.g., exons), either "from scratch" or by retaining the scaling from a previous gene-level normalization. They can easily be adapted to incorporate other sequence features such as gene length and mappability. Note, however, that in the process of adjusting for GC-content one may already be adjusting indirectly for other covariates such as length. Controls (e.g., housekeeping genes, spiked-in sequences) could also be included.

Our normalization procedures return genes-by-lanes tables of normalized counts, on the original unlogged scale and rounded to the nearest integer. Some authors have argued that it is better to leave the count data unchanged to preserve their sampling properties and instead use an *offset *for normalization purposes in the statistical model for read counts [21,22]. It is out of the scope of this article to discuss whether it is preferable to normalize counts prior to modeling or to perform normalization within the model. Nevertheless, it is worth noting that our normalization approaches can easily be modified to produce an offset, by considering the difference between normalized and unnormalized counts, in a manner similar to Hansen *et al*. [12]. The EDASeq package implements both strategies, i.e., its normalization functions can return either a table of normalized counts or a table of offsets.

We identified differentially expressed genes using a likelihood ratio test based on a negative binomial model for read counts. For the MAQC datasets, Bullard *et al*. [3] found that it was appropriate to model read counts using the Poisson distribution (negative binomial distribution with null dispersion parameter). For the Yeast dataset, substantial over-dispersion remains after both within and between-lane normalization (Figures S3 and S18), which precludes relying on the Poisson distribution. Over-dispersion is greatly reduced by between-lane normalization and much less so by within-lane GC-content normalization. The four within-lane normalization procedures seem to have similar impact on the mean-variance relationship (with slightly smaller variances for CQN), so that DE results do not appear to be driven by differences in dispersion estimates for the different procedures. Furthermore, for the Yeast dataset, goodness-of-fit analysis suggests that a negative binomial model with common dispersion parameter for the ensemble of genes is sufficient to capture the over-dispersion present in the counts (data not shown). Virtually identical results were obtained for three over-dispersion scenarios implemented in edgeR: tagwise, trended, and common dispersion. Note that the violation of Type I error control for the Yeast pseudo-datasets is actually not as serious as it might seem at first. Indeed, the largest deviations correspond to culture/library preparation effects (worst-case scenario of Figure 5) and nominal and actual Type I error rates are close for most pseudo-datasets (Figure S12). A detailed evaluation of read count models and DE methods is out-of-scope here, since our aim is to compare normalization approaches for a given DE method.

There are two different types of GC-content effects. The first effect is to act as a *proxy for sample size*, in a similar manner as length, and relates to *power*: as GC-content increases, read counts first increase then decrease, and evidence in favor of DE increases. If the effect was not sample-specific and simply a proxy for sample size, one would expect no dependence of expression fold-changes on GC-content and the effect on *p*-values to be due to the dependence of the variance on GC-content (a simple calculation can be done in the case of length and assuming counts are roughly proportional to the product of gene length and expression level). One could therefore argue that it is not justified after all to normalize for GC-content and, in particular, that FQ normalization is too aggressive. Indeed, as seen in Figure 3, within-lane normalization methods not only remove the dependence of fold-changes on GC-content, but also tend to reduce the spread of fold-changes at high GC-content (especially for FQ). This results in an overall decrease in the proportion of genes declared DE (Figures 6 and 7). Other approaches which account for GC-content could be based on standardized *p*-values, i.e., *p*-values that explicitly account for sample size [34]. A rule-of-thumb for standardizing a *p*-value *p_n _*based on a sample size of *n *to sample size 100 is ${\tilde{p}}_{n}=\min\left(\frac{1}{2},p_{n}\frac{\sqrt{n}}{10}\right)$. In lieu of the sample size *n*, one could use gene length or GC-content. The second and more insidious effect, however, is *sample-specific *and hence *biases *fold-changes and the resulting DE statistics (likelihood ratio statistics and *p*-values). In particular, the standardized *p*-value approach does not address the sample-specificity (and complexity) of the GC-content effect and would still lead to biased DE results. Likewise for methods that correct for the GC-content bias after performing DE tests, e.g., in a fashion similar to that proposed in Young *et al*. [19] for gene length bias in context of Gene Ontology analysis. We therefore find it preferable to adjust for GC-content prior to statistical modeling and DE analysis. The value of performing a within-lane GC-content normalization before combining/comparing counts between lanes is further supported by Figure 7, which shows that *p*-values based on microarray data do not vary with GC-content and hence suggests that the GC-content effect is a technology-related artifact. Of the normalization procedures we considered, full-quantile normalization seems most effective at removing the dependence of DE results on GC-content. However, results may vary in a dataset-specific manner and less aggressive approaches, such as loess or median normalization, may be robust alternatives. In the absence of controls, we recommend a thorough exploration of the data before choosing an appropriate normalization. In summary, there is a *trade-off *between bias removal and power: without within-lane GC-content normalization, fold-changes are biased, however normalization may mask true DE.

GC-content bias is even more of an issue when comparing read counts between species, e.g., allele-specific expression in diploid hybrid of *S. bayanus *and *S. cerevisiae *[9]. We are considering extensions of our methods to address GC-content bias for between-species, within-gene DE analyses.

It would also be interesting to consider adaptations of our methods to other sequencing assays, such as ChIP-Seq and DNA-Seq.

Finally, as with microarrays, positive and negative *controls *(e.g., housekeeping genes, spiked-in sequences) are essential for conclusive validation and comparison of any inference method, e.g., in terms of bias, variance, Type I error, and power. Controls could also be incorporated as "anchors" within the normalization procedure itself [35]. The use of controls from the External RNA Control Consortium (ERCC) in the recent article of Jiang *et al*. [36] is an encouraging step in this direction.

## Conclusions

We have reported the existence of strong sample-specific GC-content effects on RNA-Seq read counts, which can substantially bias differential expression analysis, and have proposed three simple within-lane gene-level GC-content normalization approaches. The GC-content effect seems to be the same for lanes assaying the same library preparation, but tends to vary between library preparations for the same type of biological sample. Hence, the bias is likely to be introduced at the library preparation step (as noted in Benjamini & Speed [6] for DNA-Seq). We have compared our methods to the state-of-the-art CQN procedure of Hansen *et al*. [12] (which was shown to outperform competing methods such as that of Pickrell *et al*. [15]), on both yeast and human RNA-Seq data, in terms of bias and mean squared error for expression fold-change estimation and in terms of Type I error and *p*-value distributions for tests of differential expression. Our proposed within-lane procedures, followed by between-lane normalization as in Bullard *et al*. [3], reduce GC-content bias and lead to more accurate estimation of expression fold-changes and tests of differential expression.

The normalization methods proposed in this article are implemented in the open-source Bioconductor R package EDASeq. The resulting normalized counts (or raw counts and associated normalization offsets) can then be supplied seamlessly to other R packages for differential expression analysis, such as DESeq [21] or edgeR [33].

## Software

The methods proposed in this article are implemented in the R package EDASeq, released as part of the Bioconductor Project http://www.bioconductor.org. This package, for exploratory data analysis and normalization for RNA-Seq, implements a variety of numerical and graphical summaries of read data, within-lane normalization procedures to adjust for GC-content or other gene-level effects, and between-lane normalization procedures to adjust for distributional differences between lanes.

## Authors' contributions

DR co-developed the statistical methods, wrote the EDASeq R package, performed data analysis, and drafted the manuscript. KS designed and performed the yeast experiments and prepared the DNA sequencing libraries. GS designed the yeast experiments and edited the manuscript. SD co-developed the statistical methods, performed data analysis, drafted the manuscript, and designed and coordinated the research. All authors read and approved the final manuscript.

## Supplementary Material

Additional file 1 **Supplementary Figures**. Additional figures referred to in the main article as Figures S1-S18. Click here for file
