# Supplementary material for: GC-Content Normalization for RNA-Seq Data
Source: BMC Bioinformatics. 2011 Dec 17;12:480. doi: 10.1186/1471-2105-12-480 (PMC3315510; doi:10.1186/1471-2105-12-480)
Supplement: Additional file 1 — Supplementary Figures. Additional figures referred to in the main article as Figures S1-S18. [file 1471-2105-12-480-S1.PDF]

Additional File 1 — Supplementary Figures

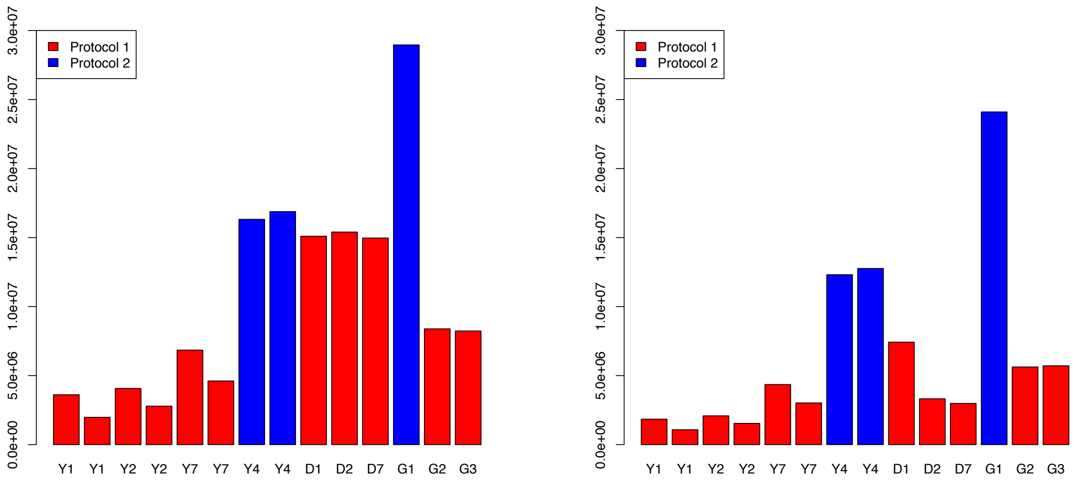

(a) Before mapping

(b) After mapping

Figure S1: *Yeast dataset: Number of reads per lane.* Barplots of total number of reads per lane, color-coded by library preparation protocol.

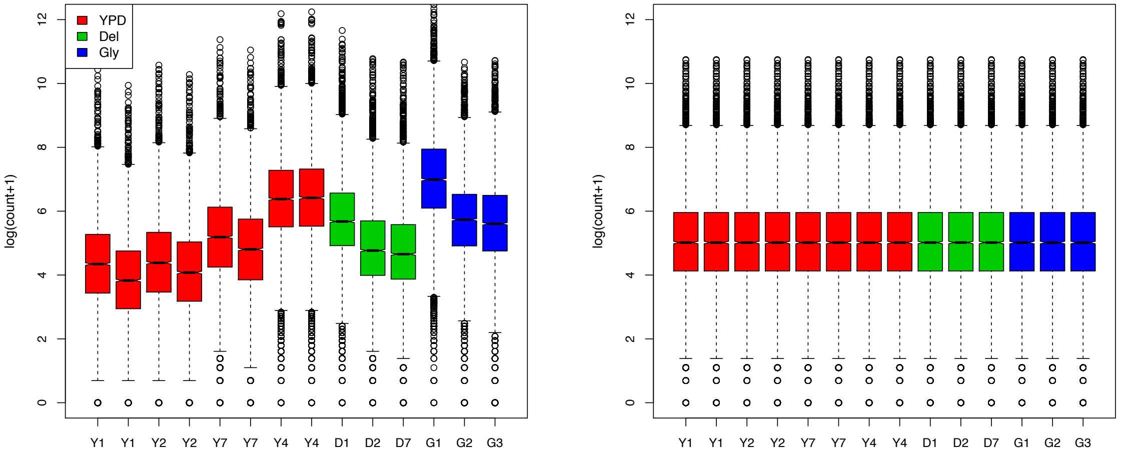

(a) Unnormalized

(b) FQ between-lane normalization

Figure S2: *Yeast dataset: Per-lane gene-level read counts.* Boxplots of gene-level log(count + 1), color-coded by growth condition. As intended after FQ between-lane normalization, the quantiles of the distributions of the gene-level counts are the same for all the lanes.

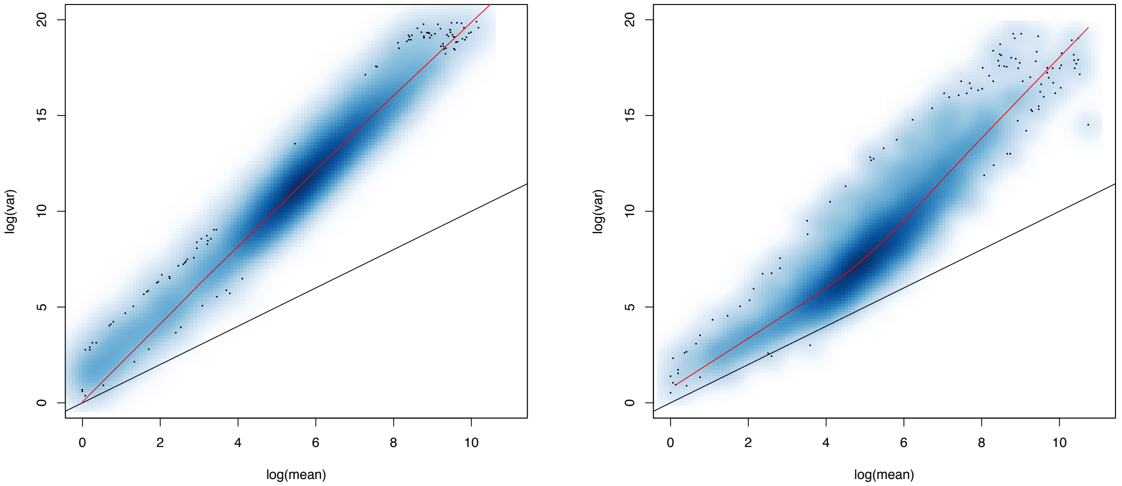

(a) Unnormalized

(b) FQ between-lane normalization

Figure S3: *Yeast dataset: Over-dispersion.* Smoothed scatterplots of log variance vs. log mean for the eight YPD lanes. Black line: Identity line (Poisson). Red curve: lowess fit. Over-dispersion is reduced by between-lane normalization, but not enough to warrant modeling gene-level counts using the Poisson distribution.

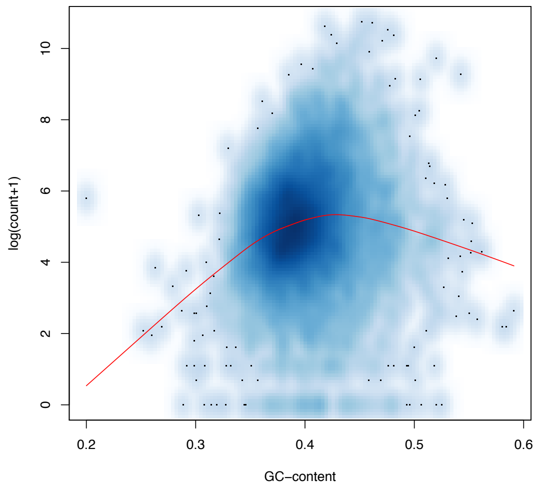

Figure S4: *Yeast dataset: Read count vs. GC-content.* Smoothed scatterplot and lowess fit of gene-level log(count + 1) vs. GC-content for the first YPD lane (culture/library preparation Y1, flow-cell 428R1), after FQ between-lane normalization.

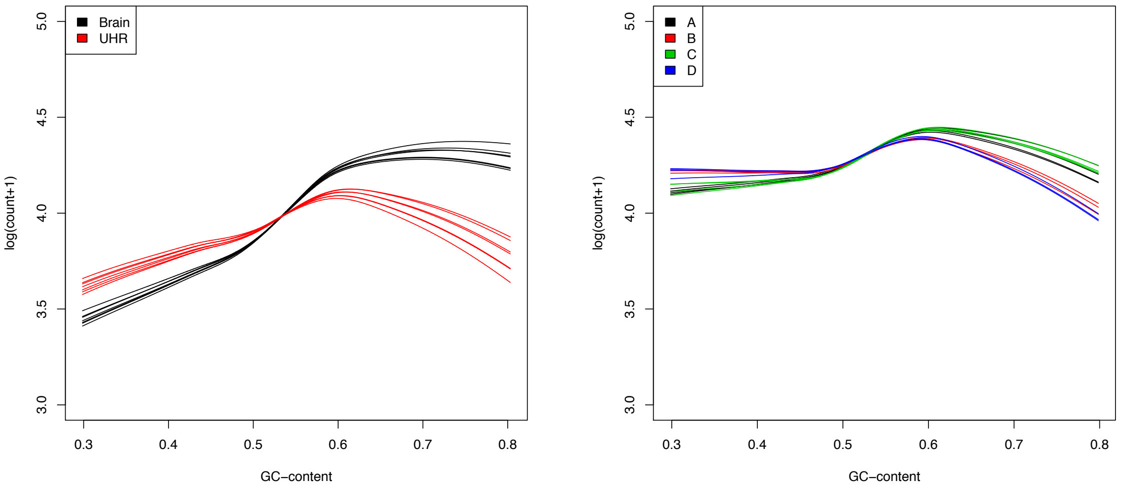

(a) MAQC-2

(b) MAQC-3

Figure S5: *MAQC datasets: Read count vs. GC-content.* Lowess fits of gene-level log(count + 1) vs. GC-content, after FQ between-lane normalization. Curves are colored according to biological sample for MAQC-2 and library preparation for MAQC-3.

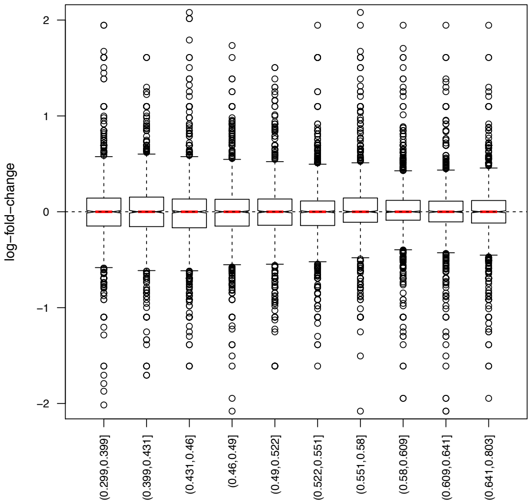

(a) Same UHR library preparation

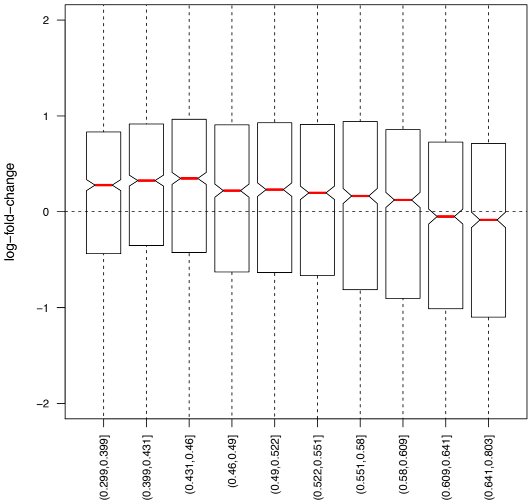

(b) UHR vs. Brain

Figure S6: *MAQC-2 dataset: Log-fold-change vs. GC-content.* Stratified boxplots of count log-ratio vs. GC-content, after FQ between-lane normalization. Panel (a): Same UHR library preparation, lanes from the same flow-cell. Panel (b): UHR lane vs. Brain lane from the same flow-cell. The GC-content effect is the same for the two lanes assaying the same UHR library preparation, so that fold-change estimates do not vary with GC-content. By contrast, the GC-content effect differs between Brain and UHR libraries and confounds fold-change estimation. Note, however, that here the extreme difference between Brain and UHR makes it difficult to assess the dependence on GC-content.

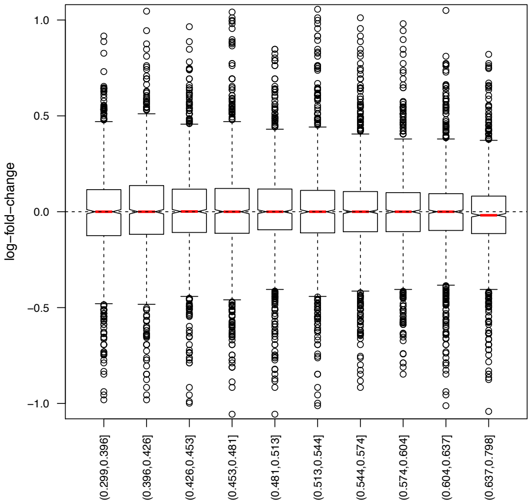

(a) Same UHR library preparation

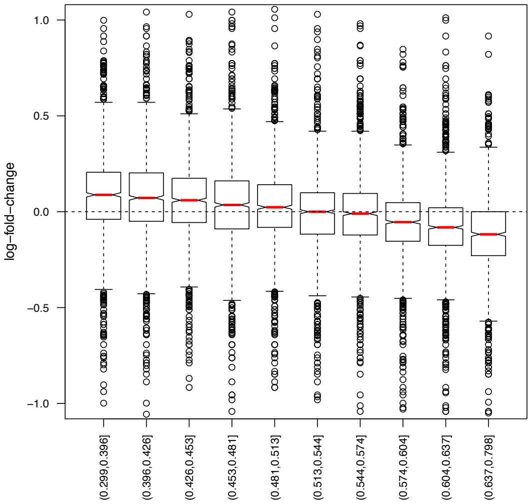

(b) Different UHR library preparations

Figure S7: *MAQC-3 dataset: Log-fold-change vs. GC-content.* Stratified boxplots of count log-ratio vs. GC-content, after FQ between-lane normalization. Panel (a): UHR library "A" lanes from the same flow-cell. Panel (b): UHR library "A" lane vs. UHR library "B" lane from the same flow-cell. The GC-content effect is the same for lanes assaying the same UHR library preparation, so that fold-change estimates do not vary with GC-content. However, the GC-content effect differs between different UHR library preparations and confounds fold-change estimation.

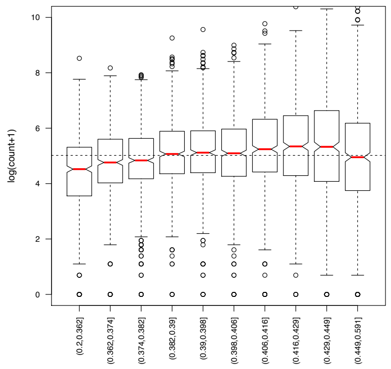

(a) Only between-lane normalization

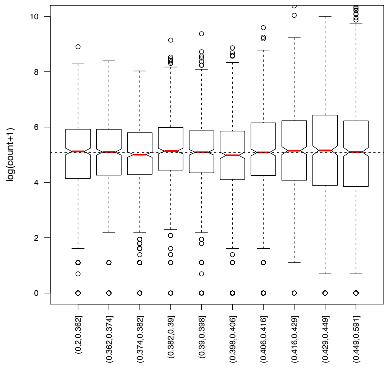

(b) loess

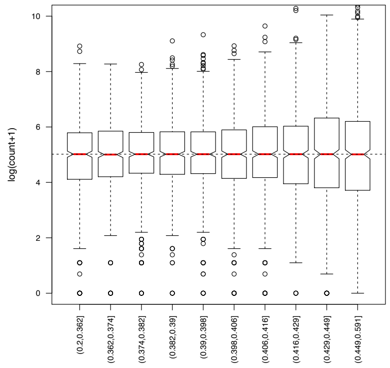

(c) Median

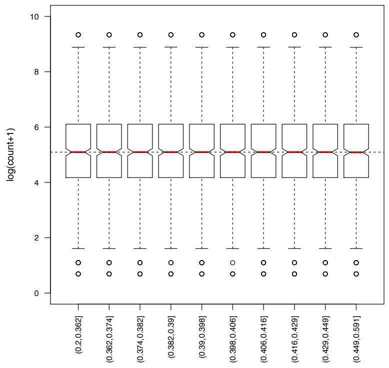

(d) FQ

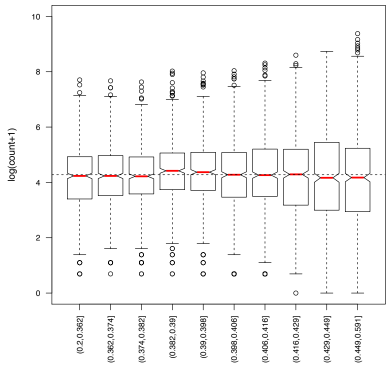

(e) CQN

Figure S8: *Yeast dataset: GC-normalized read count vs. GC-content.* Stratified boxplots of log(count + 1) vs. GC-content, for the Y1 lane of flow-cell 428R1. Panel (a): Only full-quantile between-lane normalization. Panel (b): Regression within-lane normalization using loess. Panel (c): Global-scaling within-lane normalization using the median. Panel (d): Full-quantile (FQ) within-lane normalization. Panel (e): Conditional quantile normalization (CQN). The first three within-lane procedures (Panels (b–d)) were followed by FQ between-lane normalization; CQN includes its own between-lane normalization. All within-lane methods seem to effectively reduce the dependence of counts on GC-content (compared to Panel (a)).

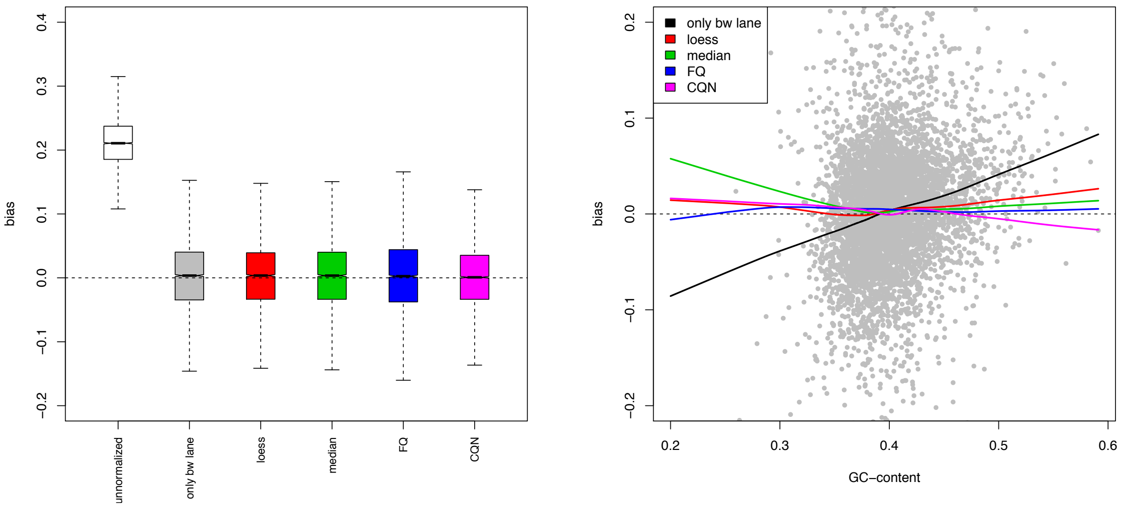

(a) Bias (b) Bias vs. GC-content

Figure S9: *Yeast YPD pseudo-datasets: Bias in fold-change estimation.* Bias in log-fold-change estimation for different normalization procedures, where, for a given gene, bias is defined as the average of the 35 log-ratios from the YPD pseudo-datasets. Panel (a): Boxplots of bias in log-fold-change estimates. Panel (b): Dependence of bias on GC-content. The points correspond to bias after only FQ between-lane normalization, the curves are lowess fits of bias vs. GC-content for different normalization procedures. All normalization procedures reduce bias, but only within-lane normalization reduces its dependence on GC-content.

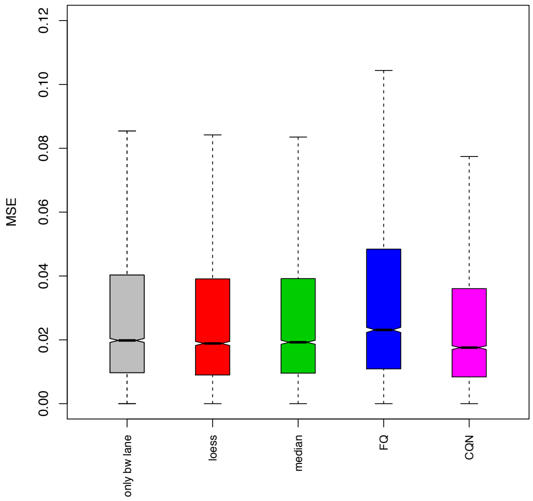

Figure S10: *Yeast YPD pseudo-datasets: Mean squared error in fold-change estimation.* Mean squared error in log-fold-change estimation for different normalization procedures, where, for a given gene, MSE is defined as the average of the square of the 35 log-ratios from the YPD pseudo-datasets.

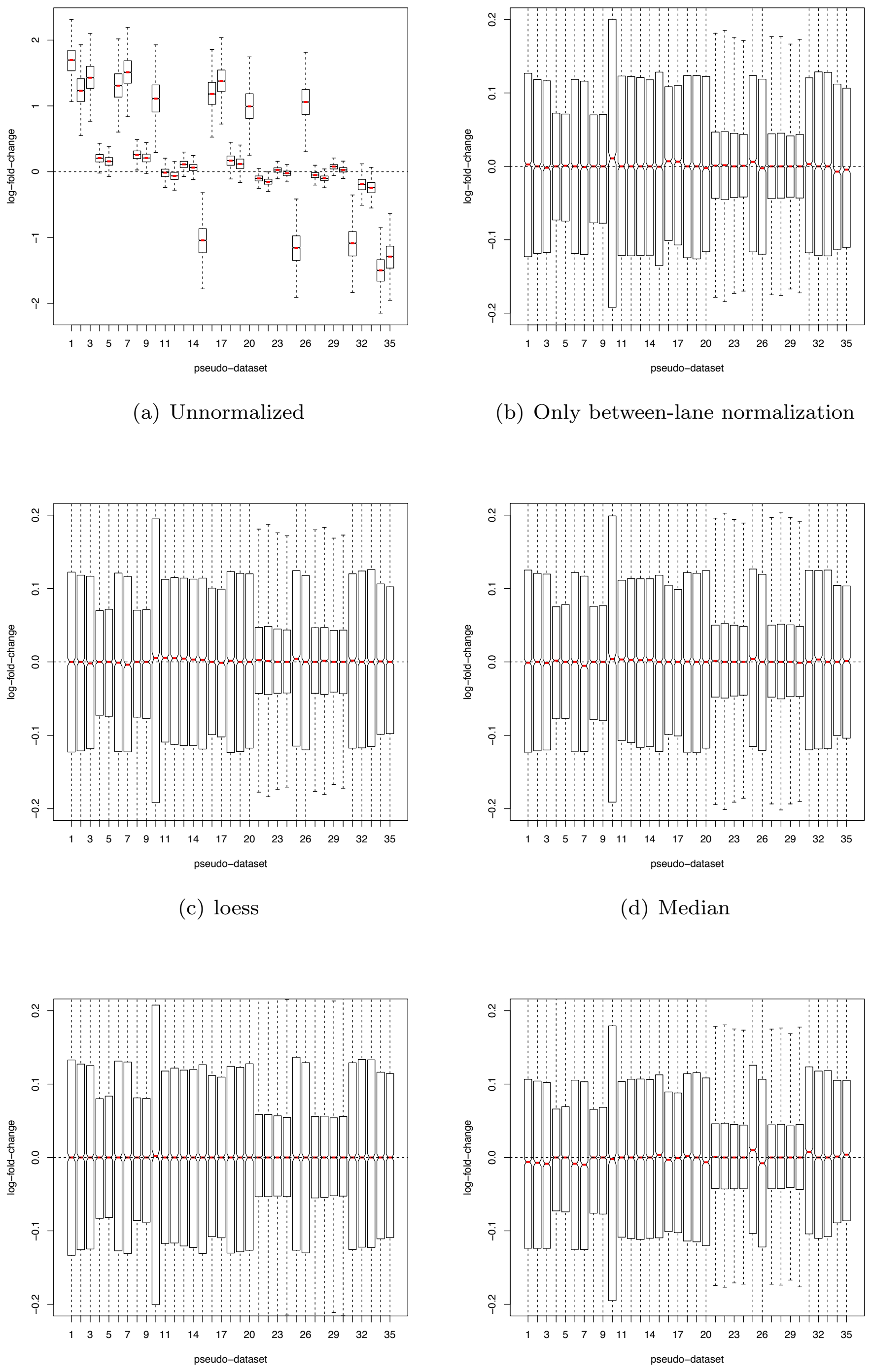

(a) Unnormalized (b) Only between-lane normalization

(c) loess (d) Median

(e) FQ (f) CQN

Figure S11: *Yeast YPD pseudo-datasets: Distribution of log-fold-changes.* Boxplots of count log-ratios for the 35 YPD pseudo-datasets, for different normalization procedures. One would expect the log-fold-changes to be around zero for each dataset. There is a clear bias for unnormalized counts, with log-fold-change estimates as high as 2 (note different scale for Panel (a)). The FQ within-lane normalization method seems to be the most coherent in estimating the log-fold-change around zero.

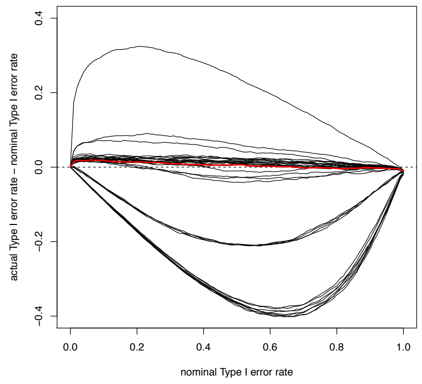

(a) Only between-lane normalization

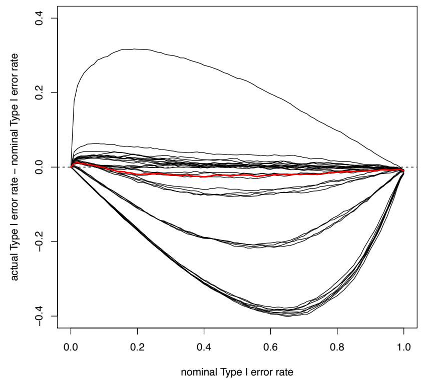

(b) loess

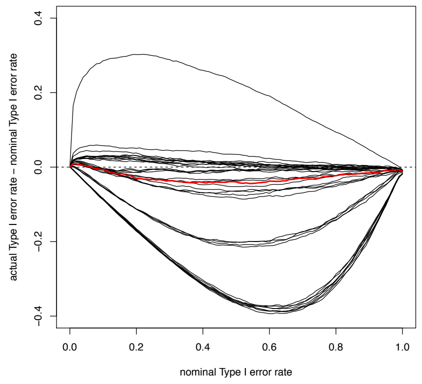

(c) Median

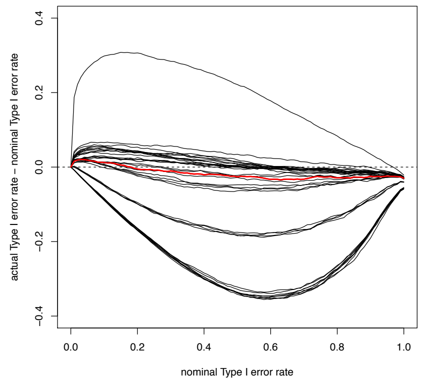

(d) FQ

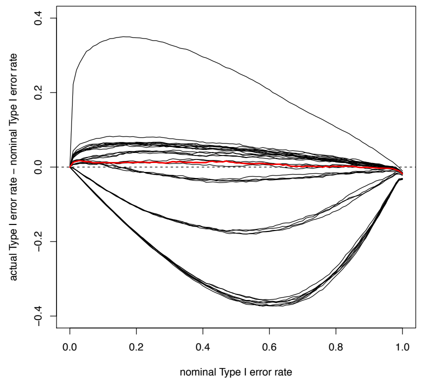

(e) CQN

Figure S12: *Yeast YPD pseudo-datasets: Type I error.* Difference between actual and nominal Type I error rates vs. nominal Type I error rate for each of the 35 YPD pseudo-datasets, for different normalization procedures. Red curve: Median difference in Type I error rates.

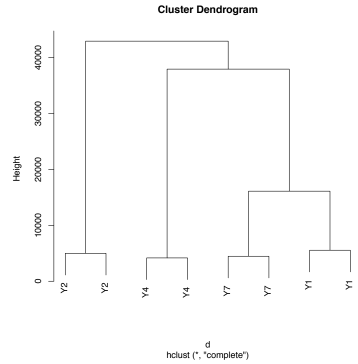

(a) Only between-lane normalization

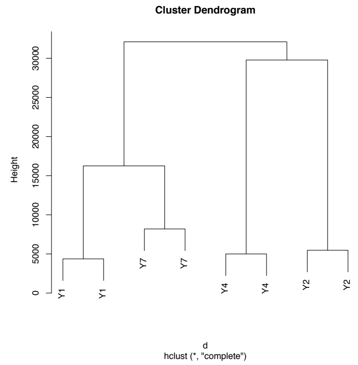

(b) loess

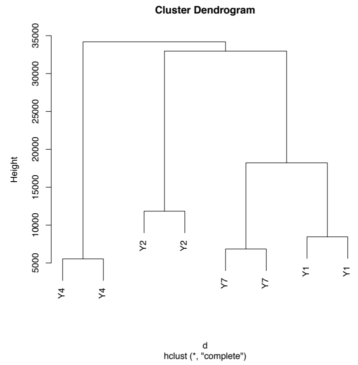

(c) Median

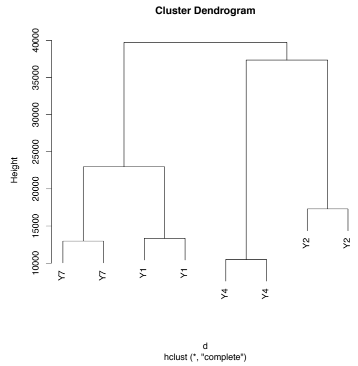

(d) FQ

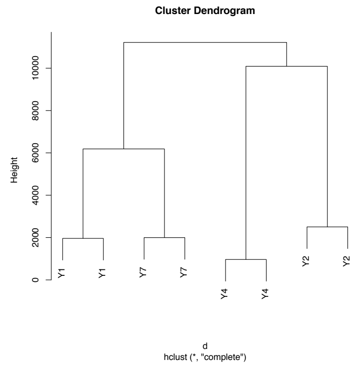

(e) CQN

Figure S13: *Yeast dataset: Hierarchical clustering of lanes.* Dendrogram for complete linkage hierarchical clustering of the eight YPD lanes, based on Euclidean distance, for different normalization procedures.

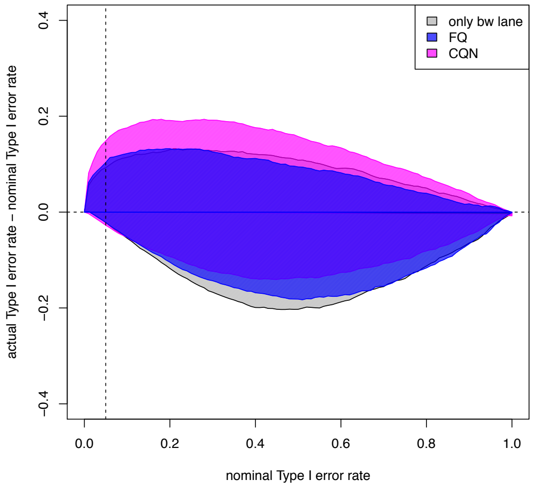

Figure S14: *Yeast YPD pseudo-datasets: Type I error.* Difference between actual and nominal Type I error rates vs. nominal Type I error rate, for different normalization procedures. The colored areas correspond to the most conservative and most anti-conservative curves obtained from the 10 YPD pseudo-datasets for libraries prepared using Protocol 1. The dashed line corresponds to a nominal unadjusted p-value of 0.05. The full-quantile GC-content normalization procedure yields the smallest area, meaning that the actual Type I error rate is closer to the nominal Type I error rate than with the other two procedures.

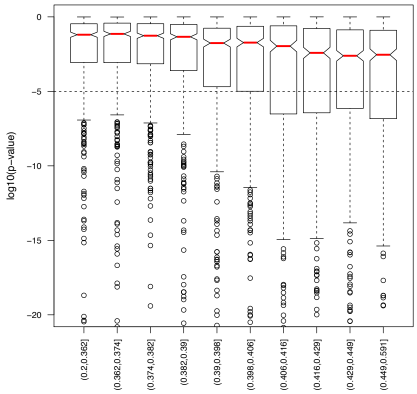

(a) Only between-lane normalization

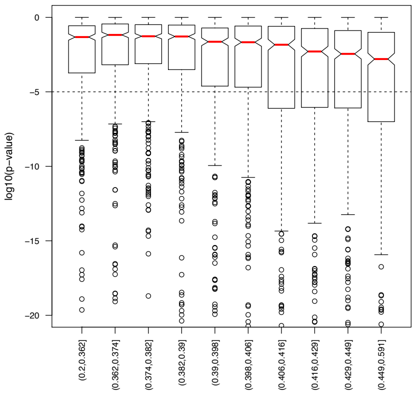

(b) loess

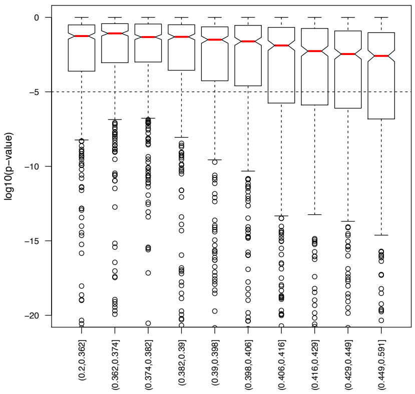

(c) Median

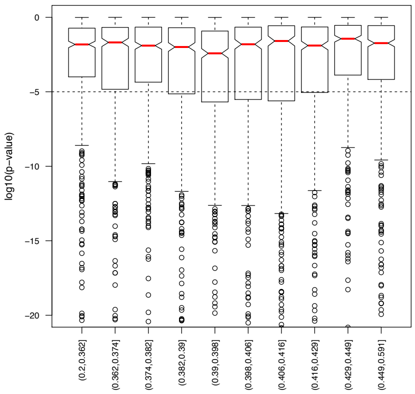

(d) FQ

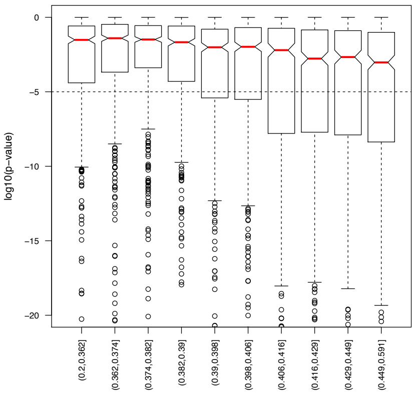

(e) CQN

Figure S15: *Yeast dataset: p-value vs. GC-content.* Stratified boxplots of unadjusted p-values ($\log_{10}$) for the negative binomial LRT of growth condition effects vs. GC-content, for different normalization procedures. As in Figure 6, for every procedure but the full-quantile, the higher the GC-content, the more significant the evidence in favor of DE. The dashed line corresponds to a nominal unadjusted p-value of 10^{-5}.

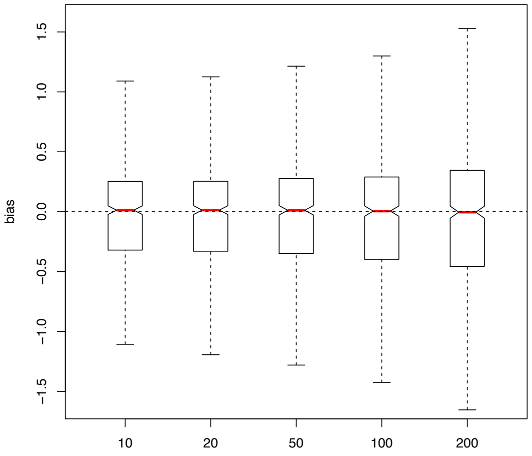

(a) MAQC-2

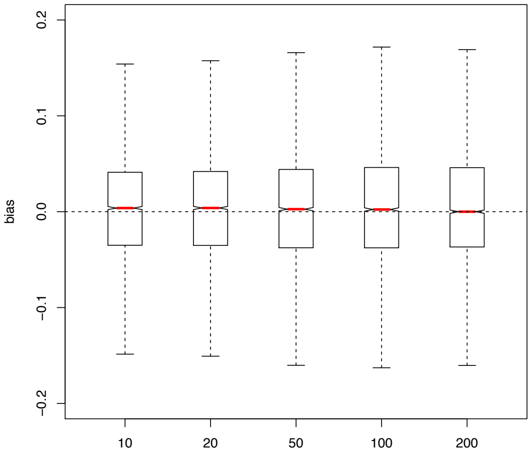

(b) Yeast YPD pseudo-datasets

Figure S16: *Bias vs. number of GC-content bins.* Boxplots of bias for MAQC-2 and Yeast datasets after full-quantile within-lane GC-content normalization with different numbers of GC-content bins. Panel (a): Estimated bias for MAQC-2 dataset, defined as difference between estimated log-fold-changes from RNA-Seq and qRT-PCR. Panel (b): Estimated bias for Yeast YPD pseudo-datasets, defined as average of the 35 log-ratios. The FQ normalization procedure appears to be robust to the number of bins.

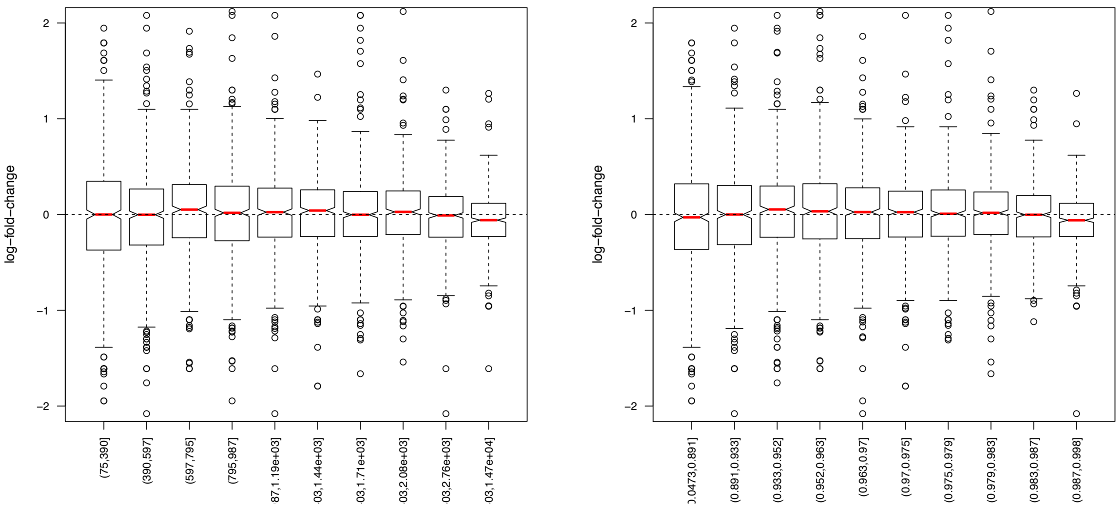

(a) Gene length (b) Gene mappability

Figure S17: *Yeast dataset: Log-fold-change vs. length and mappability.* Stratified boxplots of count log-ratios, for YPD Y1 lane vs. Y2 lane from flow-cell 428R1, after FQ between-lane normalization. Panel (a): Stratified by gene length. Panel (b): Stratified by mappability. Mappability for a given gene is defined as the ratio between the number of mappable bases and the total number of bases, where a base is said to be *mappable* if the sequence of 36 bases starting at that position is unique along the genome. Expression fold-changes do not appear to depend on either length or mappability.

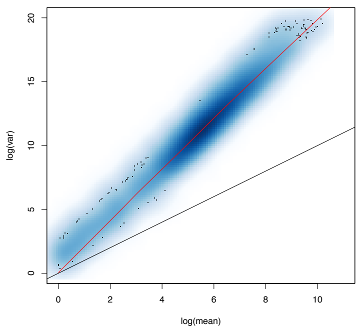

(a) Unnormalized

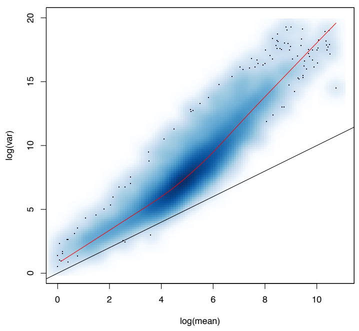

(b) Only between-lane normalization

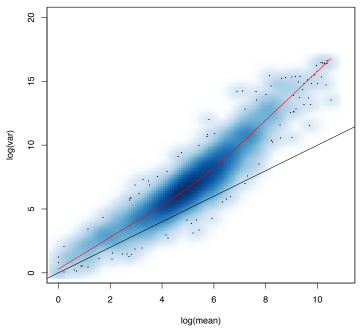

(c) loess

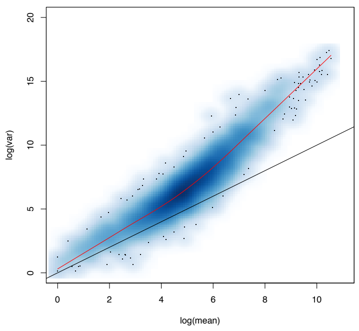

(d) Median

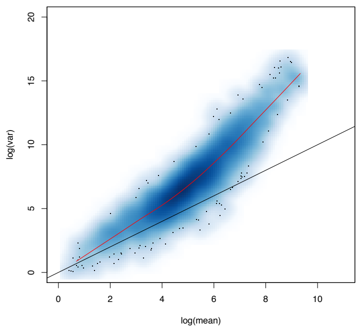

(e) FQ

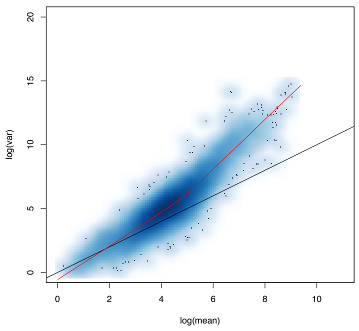

(f) CQN

Figure S18: *Yeast dataset: Over-dispersion.* Smoothed scatterplots of log variance vs. log mean for the eight YPD lanes, for different normalization procedures. Black line: Identity line (Poisson). Red curve: lowess fit. Within-lane GC-content normalization has a smaller impact than between-lane normalization on over-dispersion. CQN seems to lead to smaller variances, although results appear similar for the four within-lane normalization procedures.
